# Multi-Index Evaluation for Flood Disaster from Sustainable Perspective: A Case Study of Xinjiang in China

**DOI:** 10.3390/ijerph15091983

**Published:** 2018-09-11

**Authors:** Yudan Dou, Xiaolong Xue, Zebin Zhao, Xiaowei Luo, Ankang Ji, Ting Luo

**Affiliations:** 1 School of Management, Harbin Institute of Technology, Harbin 150001, China; douyudan@hit.edu.cn (Y.D.); xlxue@hit.edu.cn (X.X.); ankangji@stu.hit.edu.cn (A.J.); luoting@stu.hit.edu.cn (T.L.); 2 School of Management, Guangzhou University, Guangzhou 510000, China; 3 Department of Architecture and Civil Engineering, City University of Hong Kong, Hong Kong 999077, China; xiaowluo@cityu.edu.hk

**Keywords:** multi-index evaluation, combined weights, variable fuzzy recognition model, sustainable perspective

## Abstract

The floods have undermined the sustainable construction of cities because of their sudden and destruction. To reduce the losses caused by floods, it is necessary to make a reasonable evaluation for historical floods and provide scientific guidance for future precaution. Previous research mainly used subjective/objective weights or barely made static analysis without considering the uncertainty and ambiguity of floods. Therefore, this study proposed a variable fuzzy recognition model, based on combined weights, to evaluate floods, including the determination of index weights and the choice of evaluation model. To make the index weights reflect both subjective experience and objective data, the combined weights were proposed and calculated based on the principle of minimum identification information. Then, the relative membership degree matrix and evaluation results can be worked out by the variable fuzzy recognition model. Conclusions indicated that the combined weights were more convincing than simply subjective or objective weights. Moreover, the variable fuzzy recognition model, by changing model parameters, got stable evaluation results of the sample data. Therefore, the model can improve the credibility of evaluation and the conclusions can provide reasonable suggestions for management departments.

## 1. Introduction

The floods have destroyed the sustainable construction of cities, such as damaging buildings and breaking the city lifeline system, when they suddenly happened with great power [1]. The frequency of such disasters is increasing globally, according to records, and the economic losses caused by floods are often greater than other natural disasters [2]. The occurrence of floods is unpredictable, so how to prepare for floods and minimize post-disaster losses are our concerns. The flood disaster evaluation is to explore the non-linear relationship between evaluation indicators and flood levels, and to assess the damage caused by floods. Flood evaluation is fundamental work for flood precaution and mitigation, the conclusions of which can provide important guidance for disaster relief [3].

The losses caused by floods include both tangible assets, which can be quantified as money, and intangible assets. To accurately evaluate the floods level, both forms should be considered. Robert proposed a quantitative method for assessing the economic losses, according to flood scenarios, and used hydraulic models to ensure the stability of results [4]. Westen established a training package to use the 1D–2D flood propagation model for flood evaluation [5]. Ji analyzed the risk levels by taking eight atmospheric circulation indicators as independent variables, and conducted an assessment of floods in the Huaihe River through nonlinear and nonparametric classification [6]. Some researchers focused on methods and tools for evaluating floods to map, detect, and analyze flood risks [7]. The research for evaluation of flood disaster and risk generally focused on several aspects: The determination of flood disaster degree, the damage area and the influence extent [8,9], and the assessment of economic losses [10]. Schumann [11] used SAR images and the USGS Hazards Data Distribution System to describe the flood disaster in Texas [12]. Rukmana [13] developed a decision support system to determine the risk level of floods by using the fuzzy method. Kwon [14] differentiated the risk level of Twitter data and visualized on the map in order to detect signs of flood disaster as soon as possible. Hansson [15] put forward a framework for the evaluation of flood management strategies. Luino [16] studied a model for the evaluation of flood damage and described the staged damage curves.

Since the 1980s, the research on disaster sociology and disaster economics emerged rapidly, with the development of the disaster reduction activities worldwide [17]. They proposed a number of quantitative evaluation methods about the disaster losses, one after another, including the fuzzy comprehensive evaluation method [18,19,20], grey clustering analysis method [21,22], intelligent model based on artificial neural network and genetic algorithm [23,24], projection pursuit method [25], and the matter element analysis method [26], etc. Different methods can evaluate flood disasters from different angles. Those methods have some achievements and applications but there still exists certain limitations, since the index system and influence factors on flood characterized the disunity, randomness, and ambiguity. The evaluation results calculated by different methods generate incompatibility easily, which puzzled the decision makers. The simple and useful evaluation methods for flood disaster with stable and reliable results should be explored, so as to address the vague and uncertain information involved.

In view of many factors leading to floods, only using single index evaluation is unable to make an accurate analysis and support the reasonable suggestions for precaution and reduction of floods. Therefore, the assessment of floods should belong to the problem of multi-index evaluation.

The critical parts for multi-index evaluation are index weights and evaluation methods [27,28], and the index weights are always prepared for evaluation methods [29,30]. The determination of index weights and evaluation methods are generally divided into qualitative and quantitative. The qualitative evaluation method, including the Delphi method [31] and expert meeting method [32], and the quantitative evaluation method including the system engineering method [33], statistical analysis method [34,35], and the operational research method [36] etc., are the two main comprehensive evaluation methods [37]. As the appearance of mutual integration from different knowledge areas, new methods including system modeling and simulation method [38], information theory method [39], grey theory method [40,41], intelligent method [42,43,44], rough set method [45], matter element analysis method [46], and a novel MADM approach [47] for comprehensive evaluation problems emerged. Different evaluation methods suit for different research objects. It can be seen that the index weights usually considered the subjective weights or the objective weights, while few focused on both subjective and objective weights. The fuzzy mathematics method [48], in numerous evaluation methods, can overcome the disadvantages of “only one solution” and conform to the concept of “flexible management” in modern society because of scalability. Some studies have shown that the fuzzy set theory is suitable for fuzzy control, fuzzy decision making, and fuzzy mathematical programming, which are considered to be the most suitable examples to show the efficiency of the fuzzy set theory [49].

However, fuzzy set theory is a static concept, which cannot describe the dynamic variability of the fuzzy phenomena. The index system and influencing factors involved in floods are non-uniform, random, and ambiguous. In the 20th century, Chen proposed the concept of relative membership degree and relative membership functions, and established the theory of engineering fuzzy sets and variable fuzzy sets, which was a breakthrough for the static concept of the fuzzy set theory [50,51]. The variable fuzzy recognition model has achieved some results in the field of multi-index evaluation [52,53].

Therefore, the goal of this study is to make a scientific and reasonable evaluation for floods by a variable fuzzy recognition model based on combined weights, and to provide supports for government authorities on flood precaution and mitigation. In the next section, the authors will introduce the methodology for evaluating the floods. Considering the importance of weights on the results of the comprehensive evaluation [54], we compare the evaluation results of subjective weights (AHP (Analytic Hierarchy Process), binary comparison method andFCM (Fuzzy Cognitive Map)) and objective weights (entropy method and variable fuzzy method), respectively, and combine the subjective weights and objective weights using the principle of minimum identification information, and then put the combined weights to the variable fuzzy recognition model. Then, the effectiveness of the model is validated through a case study. The floods evaluation by variable fuzzy recognition model, based on combined weights, can provide supports for disaster precaution and relief decision-making. The floods distribution map can also be used for land use planning. Moreover, the model proposed by this study can be applicable to the multi-index evaluation in other fields—with a good promotion. 

## 2. Methodology

### Variable Fuzzy Recognition Model 

Variable fuzzy recognition model [55] will be demonstrated in detail as follows.

Setting *m* characteristic value vectors of the known objects to be evaluated as Equation (1).

(1) $$X=\left(x_{1},x_{2},\cdots ,x_{m}\right)$$

Making a comprehensive evaluation based on standard interval matrices of *m* indicators with *c* grades.

(2) $$I=\left[\begin{array}{ccc} {\left[a,b\right]}_{11} & {\left[a,b\right]}_{12} & \begin{array}{cc} \cdots & {\left[a,b\right]}_{1c} \end{array}\\ {\left[a,b\right]}_{21} & {\left[a,b\right]}_{21} & \begin{array}{cc} \cdots & {\left[a,b\right]}_{21} \end{array}\\ \begin{array}{c} \vdots \\ {\left[a,b\right]}_{m1} \end{array} & \begin{array}{c} \vdots \\ {\left[a,b\right]}_{m1} \end{array} & \begin{array}{c} \begin{array}{c} \vdots \end{array}\\ \begin{array}{cc} \cdots & {\left[a,b\right]}_{mc} \end{array} \end{array} \end{array}\right]=\left({\left[a,b\right]}_{ih}\right)$$

For the indicators which are the type of “bigger is better”, *a* > *b*; For the indicators which are the type of “smaller is better”, *a* < *b*. In the Equation (2), i=1,2,⋯,m;h=1,2,⋯,c.

By judging the left or right side of $M_{ih}$ which $x_{i}$ falls in, the relative membership degree of $x_{i}$ to the grade h can be calculated by the Equations (3)–(6).

When $x_{i}$ falls in the left of $M_{ih}$:

(3) $$u_{\tilde{A}}{\left(x_{i}\right)}_{h}=0.5\left[1+{\left(\frac{x_{i}-a_{ih}}{M_{ih}-a_{ih}}\right)}^{\beta }\right]x_{i}\in \left[a_{ih},M_{ih}\right]$$

(4) $$u_{\tilde{A}}{\left(x_{i}\right)}_{h}=0.5\left[1-{\left(\frac{x_{i}-a_{ih}}{M_{ih}-a_{ih}}\right)}^{\beta }\right]x_{i}\in \left[c_{ih},a_{ih}\right]$$

When $x_{i}$ falls in the right of $M_{ih}$:

(5) $$u_{\tilde{A}}{\left(x_{i}\right)}_{h}=0.5\left[1+{\left(\frac{x_{i}-b_{ih}}{M_{ih}-b_{ih}}\right)}^{\beta }\right]x_{i}\in \left[M_{ih},b_{ih}\right]$$

(6) $$u_{\tilde{A}}{\left(x_{i}\right)}_{h}=0.5\left[1-{\left(\frac{x_{i}-b_{ih}}{d_{ih}-b_{ih}}\right)}^{\beta }\right]x_{i}\in \left[b_{ih},d_{ih}\right]$$

h=1,2,⋯,l−1. In the Equations (3)–(6):$M_{ih}$ is an important parameter, setted as different values in different contexts. When $h=1,\;M_{i1}=a_{i1}$ and $h=c,\;M_{i1}=b_{ic}$. If c is an odd number, $h=l,\;M_{il}=\frac{a_{il}+b_{il}}{2}$. When h=l+1, l+2, ⋯, c, $u_{\tilde{A}}{\left(x_{i}\right)}_{h}$ should be replaced by $u_{{\tilde{A}}^{c}}{\left(x_{i}\right)}_{h}$ from the Equations (3)–(6).

According to Equations (3)–(6), the relative membership degree matrix of the object index characteristics value to each grade can be determined, and then the integrated relative membership degree of grade h can be solved using the variable fuzzy recognition model, as Equation (7). At last, make a comprehensive evaluation for the object by Equation (8) of level characteristic value.

**Definition** **1.** *Setting the contradictory fuzzy concept on domain U, the rejection properties on domain of attraction is represented by $\tilde{A}$ and ${\tilde{A}}^{c}$. Domain of attraction is a collection that is closely related to a particular attracting set. For any element u, u∈U in U, at any points in the continuum interval referred [1,0] (to $\tilde{A}$) and [0,1] (to ${\tilde{A}}^{c}$.), the relative membership degrees of attraction and rejection are $u_{\tilde{A}}\left(u\right)$ and $u_{{\tilde{A}}^{c}}\left(u\right)$, respectively, satisfying $0\leq u_{\tilde{A}}\left(u\right)\leq 1$, $0\leq u_{{\tilde{A}}^{c}}\left(u\right)\leq 1$ and $u_{\tilde{A}}\left(u\right)+u_{{\tilde{A}}^{c}}\left(u\right)$= 1. The integrated relative membership degree $V_{\tilde{A}}{\left(u\right)}_{h}^{\prime }$ of u to all grades h can be calculated by variable fuzzy recognition model, as Equation (7) [38]:*

(7) $$V_{\tilde{A}}{\left(u\right)}_{h}^{\prime }=\frac{1}{1+{\left\{\frac{\sum_{i=1}^{m}{\left[w_{i}\left(1-u_{\tilde{A}}{\left(u\right)}_{ih}\right)\right]}^{p}}{\sum_{i=1}^{m}{\left[w_{i}u_{\tilde{A}}{\left(u\right)}_{ih}\right]}^{p}}\right\}}^{\frac{\alpha }{p}}}$$

In Equation (7), $w_{i}$ is the weight of index i. There are two parameters (α,p) in Equation (7), α is the parameter as model optimization criteria, and α=1 means the minimum one power criteria, while α=2 indicates the minimum two powers criteria. p is the parameter as distance, and p=1 means the Hamming distance, while p=2 indicates the Euclidean distance. So there are four combinations of the parameters for the model, which are variable. Normalize $V_{\tilde{A}}{\left(u\right)}_{h}^{\prime }$ to $V_{\tilde{A}}{\left(u\right)}_{h}$ and applies Equation (8) to get the grade of u belonging to.

(8) $$H=\left(1,2,\cdots ,c\right)\times V_{\tilde{A}}^{T}{\left(u\right)}_{h}$$

## 3. Index Weights Calculation Methods

The accurate and reasonable weights are critical when using the variable fuzzy recognition model to make a comprehensive evaluation for the sample data. Generally, decision makers determine the weights in accordance with their subjective cognition and judgement, which has a strong individual disturbance because of different preferences and experiences for indicators despite being close to reality. In contrast, the objective weights totally depend on the sample data. If the sample data changes, the objective weights will change correspondingly. When the quality and quantity of the sample data cannot be guaranteed, simply using objective weights is inaccurate. The combined weights considering both subjective and objective weights can minimize the loss of information, which makes the evaluation results conform with objective data and are as close to the actuality as much as possible. Therefore, we determine the target weights by the combination of subjective weights and objective weights in this paper. In order to make comparative analysis more obviously, we choose AHP, binary comparison method, and FCM to solve the subjective weights, meanwhile, the entropy method and variable fuzzy method for the objective weights then form six kinds of combined weights according to the principle of minimum identification information.

### 3.1. Objective Weighting Method

#### 3.1.1. Entropy Method 

The concept of entropy created by Clausius [56], which is a measurement of disorder degree of the system, derives from thermodynamics. While the concept of information entropy proposed by Shannon in 1948 [57] describes the discrete degree of some index data. The bigger the discrete degree, the greater the influence of the indicator on evaluation results, and the bigger the index weight [58].

Calculation steps of entropy method [12]:

Step 1: Normalization of the original data. Setting the matrix of original data with m schemes and n evaluation indexes being $A={\left(a_{ij}\right)}_{m\times n}$ and normalizing it in light of the column to $R={\left(r_{ij}\right)}_{m\times n}$. $r_{ij}$ in the normalized matrix denotes the contribution of the i scheme under the indicator j.

For the indicators of “bigger is better”, the normalized formula is Equation (9):

(9) $$r_{ij}=\frac{a_{ij}-\min\left(j\right)\left\{a_{ij}\right\}}{\max\left(j\right)\left\{a_{ij}\right\}-\min\left(j\right)\left\{a_{ij}\right\}}$$

For the indicators of “smaller is better”, the normalized formula is Equation (10):

(10) $$r_{ij}=\frac{\max\left(j\right)\left\{a_{ij}\right\}-a_{ij}}{\max\left(j\right)\left\{a_{ij}\right\}-\min\left(j\right)\left\{a_{ij}\right\}}$$

Step 2: Definition of entropy. In the evaluation problem with m schemes and n indicators, the entropy of the indicator j is Equation (11):

(11) $$h_{j}=-k\overset{m}{\underset{i}{\sum }}f_{ij}\ln f_{ij}$$

$h_{j}$ indicates the total contribution of all samples to indicator $a_{j}$. In Equation (11), $f_{ij}=r_{ij}/\overset{m}{\underset{j=1}{\sum }}r_{ij}$, and the constant k=1/lnm; when $f_{ij}=0$, set $f_{ij}\ln f_{ij}=0$. From the formula, when the contribution of each scheme under some indicator tends to be uniform, h converges to 1; in particular, when they are all equal, the weight of the indicator is zero.

Thus, the weights of indexes are determined by the value of differences among all schemes, then defining $d_{j}$ to be the degree of consistency of contribution of each scheme under the indicator j, $d_{j}=1-h_{j}$.

Step 3: Calculation of the entropy weights. The entropy weight of the indicator j can be solved by Equation (12).

(12) $$w_{j}=d_{j}/\sum_{j=1}^{m}d_{j}\left(0\leq w_{j}\leq 1,\sum_{1}^{n}w_{j}=1\right)$$

When $d_{j}=0$, the indicator j whose weight is zero, can be deleted.

#### 3.1.2. Variable Fuzzy Method 

The derivation process of variable fuzzy model [55] computing objective weights is as follows.

Firstly, determining the initial solution of the target weights vector, and at this time the target weights $w_{i}$ and $u_{hj}$ are both unknown. According to Equation (13), the difference between scheme j and grades 1,2,⋯,c can be represented by the weighted generalized Euclidean weighted distance quadratic sum.

(13) $$f_{j}\left(u_{j}^{\bar{w}},w^{\bar{w}}\right)=\overset{c}{\underset{h=1}{\sum }}{\left\{u_{hj}\sqrt{\overset{m}{\underset{i=1}{\sum }}{\left[w_{i}\left(r_{ij}-s_{h}\right)\right]}^{2}}\right\}}^{2}$$

For the schemes sets to be the optimized as Equation (14).

(14) $$f\left(u^{\bar{w}},w^{\bar{w}}\right)=\left[f_{1}\left(u_{1}^{\bar{w}},w^{\bar{w}}\right),f_{2}\left(u_{2}^{\bar{w}},w^{\bar{w}}\right),\cdots ,f_{n}\left(u_{n}^{\bar{w}},w^{\bar{w}}\right)\right]$$

Obviously, the smaller $f_{j}\left(u_{j}^{\bar{w}},w^{\bar{w}}\right)$ is, the smaller the difference of the scheme j to grade h is, that is, the better the recognition to grade h is. So the optimization problems of multi-objective decision-making for unlimited schemes can be established, and the objective function is shown as Equation (15):

(15) $$\min\left\{f\left(u^{\bar{w}},w\right)\right\}$$

(16) $$s.t.\{\begin{array}{c} \overset{m}{\underset{i=a}{\sum }}w_{i}=1,\;\forall j\\ \overset{c}{\underset{h=1}{\sum }}u_{hj}=1,\;\forall j \end{array}$$

All the schemes in schemes sets are on equal status and there is no emphasis. Making use of the linear weighted average method with the same weights for schemes to solve the objective function, and transferring the multi-objective decision-making optimization problem into a single objective optimization problem, just as follows:

(17) $$\min\left\{F\left[u^{\bar{w}},w^{\bar{w}}\right]=\overset{n}{\underset{j=1}{\sum }}f_{j}\left[u^{\bar{w}},w^{\bar{w}}\right]\right\}$$

It should satisfy the same constraint conditions as Equation (16).

Aiming at the single objective optimization problem, constructing the Lagrangian function:

(18) $$\begin{array}{cc} L\left[w^{\bar{w}},w^{\bar{w}},\lambda_{1},\lambda_{2}\right] & =\overset{n}{\underset{j=1}{\sum }}f_{j}\left[u_{j}^{\bar{w}},w^{\bar{w}}\right]-\lambda_{1}\left[\overset{c}{\underset{h=1}{\sum }}u_{hj}-1\right]-\lambda_{2}\left[\overset{m}{\underset{i=1}{\sum }}w_{i}-1\right]\\ & =\overset{n}{\underset{j=1}{\sum }}\overset{c}{\underset{h=1}{\sum }}\left\{u_{hj}^{2}\overset{m}{\underset{i=1}{\sum }}{\left[w_{i}\left(r_{ij}-s_{h}\right)\right]}^{2}\right\}\lambda_{1}\left[\overset{c}{\underset{h=1}{\sum }}u_{hj}-1\right]-\lambda_{2}\left[\overset{m}{\underset{i=1}{\sum }}w_{i}-1\right] \end{array}$$

(19) $$\frac{\partial L}{\partial u_{hj}}=0\;\frac{\partial L}{\partial w_{i}}=0\;\frac{\partial L}{\partial \lambda_{1}}=0\;\frac{\partial L}{\partial \lambda_{1}}=0$$

The iterative model of objective weights can be obtained from Equations (20) and (21), i=1,2,⋯,m;j=1,2,⋯,n;h=1,2,⋯,c.

(20) $$u_{hj}=\frac{1}{\sum_{k=1}^{c}\frac{\sum_{i=1}^{m}{\left[w_{i}\left(r_{ij}-s_{h}\right)\right]}^{2}}{\sum_{i=1}^{m}{\left[w_{i}\left(r_{ij}-s_{k}\right)\right]}^{2}}}$$

(21) $$w_{i}=\frac{1}{\sum_{l=1}^{c}\frac{\sum_{j=1}^{n}\sum_{h=1}^{c}{\left[u_{hj}\left(r_{ij}-s_{h}\right)\right]}^{2}}{\sum_{j=1}^{n}\sum_{h=1}^{c}{\left[u_{hj}\left(r_{ij}-s_{h}\right)\right]}^{2}}}$$

The specific iterative steps are shown as follows:

Step 1: Given that the iteration tolerance of $w_{i}$ is ε;

Step 2: Setting the goal initial weights vector at random $w^{0}=\left[w_{1}^{0},w_{2}^{0},\cdots ,w_{m}^{0}\right],\;\overset{m}{\underset{i=1}{\sum }}w_{i}^{0}=1$;

Step 3: Putting $w^{0}$ into Equation (20), and solving the corresponding initial matrix $\left(u_{hj}^{0}\right)$;

Step 4: Putting the matrix $\left(u_{hj}^{0}\right)$ into Equation (21) to solve vector $w^{1}$. Comparing $w^{1}$ and $w^{0}$, and if $\max\left|w_{i}^{1}-w_{i}^{0}\right|\leq \varepsilon$, then the iteration ends and the final results can be calculated. Otherwise, continuing the iteration to l times. Because Equations (20) and (21) are proved to be convergent in theory, it will be able to meet the scheduled accuracy, namely $\underset{i}{\max}\left|w_{i}^{l}-w_{i}^{l-1}\right|\leq \varepsilon$.

### 3.2. Subjective Weighting Method

#### 3.2.1. Analytic Hierarchy Process

Analytic Hierarchy Process (AHP) was proposed by professor T L Saaty in the 1970s [59,60], and its specific algorithm steps are shown as follows:

Step 1: Establishment of the hierarchical structure model.

Step 2: Construction of the judgment matrix. Comparing the relative importance of every two indicators in the same grade to get the ratio of relative weights, on the basis of which constructing the judgment matrix A, $a_{ij}$ means the ratio of the relative weight between the indicator i and j. There are two common patterns of judgment matrix scale, including the 1~9 scale method and the index scale method. This paper selects the 1~9 scale method in light of isotonicity and handleability, as shown in Table 1.

(22) $$A=\left[\begin{array}{ccc} 1 & \frac{\omega_{1}}{\omega_{2}} & \begin{array}{cc} \cdots & \frac{\omega_{1}}{\omega_{n}} \end{array}\\ \frac{\omega_{2}}{\omega_{1}} & 1 & \begin{array}{cc} \cdots & \frac{\omega_{2}}{\omega_{n}} \end{array}\\ \begin{array}{c} \vdots \\ \frac{\omega_{n}}{\omega_{1}} \end{array} & \begin{array}{c} \vdots \\ \frac{\omega_{n}}{\omega_{2}} \end{array} & \begin{array}{cc} \begin{array}{c} \;\\ \cdots \end{array} & \begin{array}{c} \vdots \\ 1 \end{array} \end{array} \end{array}\right]=\left[\begin{array}{ccc} \alpha_{11} & \alpha_{12} & \begin{array}{cc} \cdots & \alpha_{1n} \end{array}\\ \alpha_{21} & \alpha_{22} & \begin{array}{cc} \cdots & \alpha_{2n} \end{array}\\ \begin{array}{c} \vdots \\ \alpha_{n1} \end{array} & \begin{array}{c} \vdots \\ \alpha_{n2} \end{array} & \begin{array}{cc} \begin{array}{c} \;\\ \cdots \end{array} & \begin{array}{c} \vdots \\ \alpha_{nn} \end{array} \end{array} \end{array}\right]$$

Step 3: Calculation of the weights using the arithmetic average method. 

Normalizing each column vector of the judgment matrix A, and then getting the row vector by summarizing the row data.

(23) $$\bar{W_{i}}=\overset{n}{\underset{j=1}{\sum }}\bar{a_{ij}}\left(i=1,2,\ldots ,n\right)$$

Step 4: Consistency check. Calculating the largest eigenvalue of matrix A, and then working out the consistency indicator and consistency ratio. When CR<0.1, the consistency of judgment matrix is regarded to be acceptable. The corresponding values of RI are listed in Table 2. 

Consistency indicator and consistency ratio: $CI=\frac{\lambda_{\max}-n}{n-1}$, $CR=\frac{CI}{RI}$.

#### 3.2.2. Binary Comparison Method 

The calculation process of binary comparison method [61] will be elaborated as follows.

In the limited schemes and target decisions, noting the decision set $D=\left\{d_{1},d_{2},\cdots ,d_{i},\cdots ,d_{n}\right\},\;i=1,2,\cdots n$, the target set of each scheme $O=\left\{o_{1},o_{2},\cdots ,o_{k},\cdots ,o_{m}\right\},\;k=1,2,\cdots m$, the weight vector of the target $\omega =\left\{\omega_{1},\omega_{2},\cdots ,\omega_{m}\right\}$, and the set of the target value k in decisions set $D_{k}=\left\{{}_{k}d_{1},{}_{k}d_{2},{}_{k}d_{i},\cdots ,\cdots ,{}_{k}d_{n}\right\}$.

Making sure of all targets in targets set O on importance using the binary comparison method [38], and getting the ordering results of m targets on the importance degree, which subject to sorting consistency, we suppose $o_{1}≻o_{2}≻\cdots ≻o_{m}$.

**Definition** **2.** *Making a binary comparison for $o_{k}$ and $o_{l}$(k=1,2,⋯,m,l=1,2,⋯m) on the importance degree in the targets set O.*

*Step* *1:* *When $o_{k}$ is more important than $o_{l}$, $0.5<\beta_{kl}\leq 1$;*
*Step* *2:* *When $o_{l}$ is more important than $o_{k}$, $0\leq \beta_{kl}<0.5$ and $\beta_{kl}=1-\beta_{ik}$;*
*Step* *3:* *When $o_{k}$ is as important as $o_{l}$, $\beta_{kl}=0.5$, in particular, $\beta_{kk}=0.5$.*

*We call $\beta_{kl}$ fuzzy scale value of relative importance between adjacent target $o_{k}$ and $o_{l}$. Particularly, setting the sort of targets on importance $o_{1}≻o_{2}≻\cdots ≻o_{m}$ and calling $\beta_{k_{1},k_{1}+1}\left(k_{1}=1,2,\cdots ,m-1\right)$ fuzzy scale value of relative importance for adjacent targets.*

The value of $\beta_{kl}$ can be get by tone operator table [38] of the importance degree between target $o_{k}$ and $o_{l}$.

Through the derivation, the formula of calculating the target weights is obtained as follows:

(24) $$\omega_{k}=2\sum_{l=1}^{m}\beta_{kl}/m\left(m-1\right)k,l=1,2,\cdots m,k\neq l$$

#### 3.2.3. Fuzzy Cognitive Map 

Fuzzy cognitive map (FCM), proposed by Kosko (1986) [62], is dynamic system analysis and modeling approach through causal reasoning based on the cognitive map method [63] and the fuzzy set theory [64]. FCM combines both fuzzy logic and neural networks, which indicates intuitive expression and good reasoning skills [65]. Therefore, FCM can be used to simulate complex and dynamic systems.

The feature of FCM is that the causal relationship between nodes is fuzzy and there is a dynamic feedback mechanism. The reasoning process of FCM mainly depends on the adjacency matrix composed on weights and the state matrix of nodes, which will be introduced specifically as follows.

First, setting the concept set of FCM $N=\left\{N_{1},N_{2},\cdots N_{n}\right\}$, where $N_{i}$ indicates the i concept or attribute. W is the adjacency matrix which reflects the interaction between concepts. C is the state matrix of the concepts, where $C^{\left(0\right)}$ represents the initial state vector and $C^{\left(t\right)}$ is the state vector of the t iteration. f(x), ensuring that the outputs of each iteration are in the interval of [0,1], which is the threshold function. This paper uses the following threshold function,

(25) $$f\left(x\right)=\tanh\left(x\right)=\left(1-e^{-x}\right)\left(1+e^{-x}\right)$$

The hyperbolic tangent function f(x)=tanh(x) is one of the commonly used threshold functions, which can normalize the inputs of the concept nodes and permit the value of the nodes to be negative.

The interaction between a specific concept and other concepts is calculated by the iterative formula.

(26) $$C^{\left(t+1\right)}=f\left(C^{\left(t\right)}W\right),C^{\left(0\right)}=I_{n\times n}$$

After a certain number of iterations, if the state value of the concept nodes reaches one of the following three conditions, it is considered that a steady state has been reached, and the iteration is ended. Specifically, (a) the state value is stable at a fixed value, (b) the change in state value is periodic, and (c) presenting a chaotic state, that is, the state value is uncertain and random.

Calculation steps of FCM are shown as follows.

Step 1, getting the local weight vector by the eigenvalue method.

Step 2, depicting the FCM map to reflect the interaction between concepts.

Step 3, getting a steady state matrix by iteration according to Equation (1).

Step 4, calculating the overall weight vector. To obtain the overall weight, the local weight vector (z) and the steady state matrix $\left(C^{*}\right)$ should be normalized as follows.

(27) $$Z_{n}=\frac{1}{\lambda }z$$

where, λ is the largest element in the vector z and γ is the largest row sum in the matrix C.

(28) $$C_{n}^{*}=\frac{1}{\gamma }C^{*}$$

Then, the overall vector can be got.

(29) $$w=Z_{n}+C_{n}^{*}Z_{n}$$

FCM has two obvious shortcomings: Strong dependence on expert opinions and the final state may converge to the state outside the expectation. To enhance the validity and robustness of FCM, the learning algorithm is necessary for updating the weight matrix. This study uses Nonlinear Hebbian Learning rules (NHL) [66].

### 3.3. Combined Weighting Method

Combination of subjective and objective weights, in general, is determined by linear combination method which lacks explanatory ability. This paper adopts the following method:

Suppose the objective weights vector calculated by the entropy method is $w_{1}$ (the objective weights vector calculated by the variable fuzzy method is $w_{1}^{\prime }$), the subjective weights vector by AHP is $w_{2}$ (the subjective weights vector calculated by the binary comparison method is $w_{2}^{\prime }$ and the subjective weights vector calculated by FCM is $w_{2}^{\prime \prime }$), and the combined weights vector is w. According to the minimum identification information principle [67], the following objective function is established in order to make the combined weights be as close to the reality as much as possible:

(30) $$\min\;F=\sum_{i=1}^{m}w\left(i\right)\left[\ln\frac{w\left(i\right)}{w_{1}\left(i\right)}\right]+\sum_{i=1}^{m}w\left(i\right)\left[\ln\frac{w\left(i\right)}{w_{2}\left(i\right)}\right]$$

(31) $$s.t.\;\sum_{i=1}^{m}w\left(i\right)=1;\;w\left(i\right)>0$$

Calculate the function by Lagrange multiplier method, and the combined weights can be solved as follows:

(32) $$w\left(i\right)=\frac{{\left[w_{1}\left(i\right)w_{2}\left(i\right)\right]}^{0.5}}{\sum_{i=1}^{m}{\left[w_{1}\left(i\right)w_{2}\left(i\right)\right]}^{0.5}}$$

Put the combined weights into Equation (7), and calculate the integrated relative membership degree $V_{\tilde{A}}{\left(u\right)}_{h}^{\prime }$ at all grades, and then normalize them to uua. In light of Equation (8), calculate the disaster grades where the samples can be.

## 4. Case Study

Flood evaluation is a fuzzy problem and the evaluation criterion is usually an interval, which means it is suitable for fuzzy recognition. This paper quotes the data example from Reference [68] to make an analysis and verification, targeting the flood disaster evaluation index system, and realizes recognition and evaluation by the variable fuzzy recognition model. 

The original data are listed in Table 3.

### 4.1. Objective Weights Results

Since all the indicators are the type “smaller is better”, we normalize the original data by the Equation (10) as shown in Table 4.

In accordance with Equation (12), the objective weights vector calculated by the entropy method using Matlab programming is $w_{1}=\left(0.1596,0.2032,0.2524,0.3848\right)$.

In light of Equations (20) and (21), the objective weights vector calculated by the variable fuzzy method using Matlab programming is $w_{1}^{\prime }=\left(0.4834,0.2116,0.1486,0.1564\right)$, with the initial weights vector $w_{0}^{\prime }=\left(0.25,0.25,0.25,0.25\right)$ and the target accuracy ε=0.0001.

### 4.2. Subjective Weights Results

According to the formula of AHP, the consistency indicator can be solved by using Matlab programming, CR=0.0038<0.1, which satisfies the consistency test. The subjective weights vector calculated by AHP is $w_{2}=\left(0.1093,0.3508,0.1892,0.3507\right)$.

In a similar way, the subjective weights vector calculated by binary comparison method is $w_{2}^{\prime }=\left(0.2210,0.3310,0.1780,0.2790\right)$. 

In order to validate the influence of individual preference on the subjective weights, combined weights and the evaluation results, we choose two groups of experts to fill in the judgment matrix for AHP and the binary comparison method separately.

**Figure 1:**
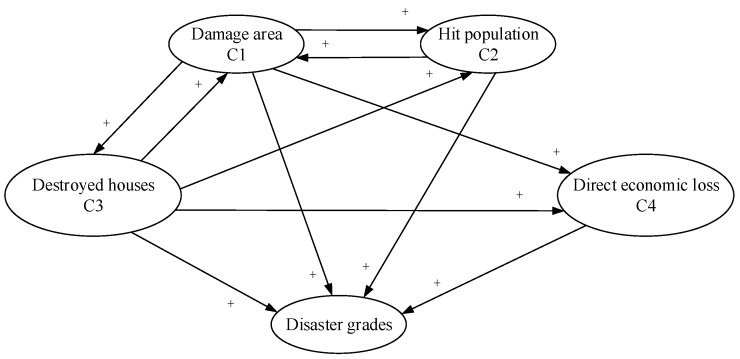
Evaluation model of floods based on Fuzzy Cognitive Map.

The reasoning process of FCM combines fuzzy logic and neural network, which has more advantages in comparison with AHP and binary comparison method because it considers fuzziness and the interaction between indexes. Firstly, the authors established a FCM model, shown in Figure 1. Where, $C_{1}$, $C_{2}$, $C_{3}$ and $C_{4}$ indicate the damage area, hit population, destroyed houses, and direct economic loss, respectively. “+” represents that the interaction between concepts as positive.

The normalized local weights calculated by the eigenvalue method are.

$$w_{L}=\left[w_{C1},w_{C2},w_{C3},w_{C4}\right]=\left[0.1091,0.3509,0.1890,0.3509\right]$$

The initial weight matrix can be obtained by experts in the disaster evaluation field, as follows.

$$W_{0}=\left[\begin{array}{cccc} 0.0000 & 0.4500 & 0.4500 & 0.5000\\ 0.2500 & 0.0000 & 0.0000 & 0.0000\\ 0.4500 & 0.5200 & 0.0000 & 0.4800\\ 0.0000 & 0.0000 & 0.0000 & 0.0000 \end{array}\right]$$

Training the weight matrix using the Hebbian learning algorithm, setting learning efficiency parameter to be 0.01 and weight attenuation factor to be 0.95, and the steady matrix can be got as follows:

$$W=\left[\begin{array}{cccc} 0.0000 & 0.0919 & 0.0894 & 0.0923\\ 0.0903 & 0.0000 & 0.0000 & 0.0000\\ 0.0899 & 0.0907 & 0.0000 & 0.0904\\ 0.0000 & 0.0000 & 0.0000 & 0.0000 \end{array}\right]$$

The overall weights of the concepts (indexes) can be obtained according to the formula.

$$w_{2}^{\prime \prime }=\left(0.4071,0.3869,0.4571,0.3509\right)$$

### 4.3. Combined Weights Results

Calculating the six kinds of combined weights by Equation (32), using the principle of minimum identification information, the results are shown in Table 5.

### 4.4. Variable Fuzzy Evaluation 

Both grades indexes and disaster grades of the flood evaluation system are shown in Table 6.

We put the values of all indicators of 10 regions or 10 schemes into the evaluation model, and calculated the corresponding flood disaster grades by the variable fuzzy recognition model. This paper randomly selects the data of Kezhou (scheme 8) as an example to elaborate the evaluation process, that is, the value sector of indicators is $x_{i}=\left(0.341,5.600,1.556,0.395\right)$. Make use of the variable fuzzy recognition model based on the combined weights to evaluate the sample data as the following steps.

Firstly, we construct the value matrixes for all parameters (a,b,c,d,M) of the variable fuzzy evaluation model, and then get the following domain of attraction $I_{ab}$, range domain $I_{cd}$, and the value of M on the basis of the variable fuzzy evaluation theory and method.

$$I_{ab}=\left[\begin{array}{ccccc} \left[0.003,0.01\right] & \left[0.01,0.1\right] & \left[0.1,1\right] & \left[1,10\right] & \left[10,60\right]\\ \left[0.003,0.01\right] & \left[0.01,0.1\right] & \left[0.1,1\right] & \left[1,10\right] & \left[10,60\right]\\ \left[0.003,0.01\right] & \left[0.01,0.1\right] & \left[0.1,1\right] & \left[1,10\right] & \left[10,60\right]\\ \left[0.003,0.01\right] & \left[0.01,0.1\right] & \left[0.1,1\right] & \left[1,10\right] & \left[10,60\right] \end{array}\right]$$

$$I_{cd}=\left[\begin{array}{ccccc} \left[0,0.1\right] & \left[0.003,1\right] & \left[0.01,10\right] & \left[0.1,30\right] & \left[1,60\right]\\ \left[0,0.1\right] & \left[0.003,1\right] & \left[0.01,10\right] & \left[0.1,30\right] & \left[1,60\right]\\ \left[0,0.1\right] & \left[0.003,1\right] & \left[0.01,10\right] & \left[0.1,30\right] & \left[1,60\right]\\ \left[0,0.1\right] & \left[0.003,1\right] & \left[0.01,10\right] & \left[0.1,30\right] & \left[1,60\right] \end{array}\right]$$

$$M=\left[\begin{array}{ccccc} 0.003 & 0.01 & 0.55 & 10 & 60\\ 0.003 & 0.01 & 0.55 & 10 & 60\\ 0.003 & 0.01 & 0.55 & 10 & 60\\ 0.003 & 0.01 & 0.55 & 10 & 60 \end{array}\right]$$

In light of the direction (left or right) where the matrix and judgment evaluation index lying to the point M, we calculated the relative membership grade of various rank standards $u_{\tilde{A}}{\left(u\right)}_{mh}$ as follows:

$$u_{\tilde{A}}{\left(u\right)}_{mh}=\left[\begin{array}{ccccc} 0.0000 & 0.3661 & 0.7678 & 0.1339 & 0.0000\\ 0.0000 & 0.0000 & 0.2444 & 0.7556 & 0.2556\\ 0.0000 & 0.0000 & 0.4691 & 0.5309 & 0.0309\\ 0.0000 & 0.3361 & 0.8278 & 0.1639 & 0.0000 \end{array}\right]$$

**Figure 2:**
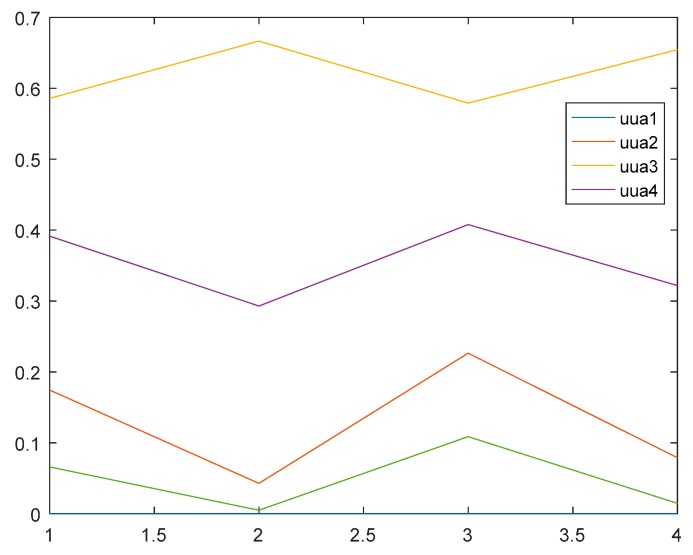
The comprehensive relative membership degree curves of all indicators for different grades of disaster.

The authors put the weights values into Equation (7) and changed the corresponding combinations of different parameters, and then the integrated relative membership degrees of indicators can be obtained by programming in matlab2010a, as shown in Figure 2.

From the top to the root of the figure, the first line is the integrated relative membership degree of indicators related to the third grade, and the purple one is the comprehensive relative membership degree related to the fourth grade, and the rest can be dealt with in the same manner. Then it is easy to see that the maximum relative membership degree of indicators for different levels is the third grade, and the flood disaster grade of Kezhou can be determined as the third grade, according to the principle of maximum degree of membership, that belongs to moderate disaster specifically. Further confirmation by variable fuzzy numerical calculation is subsequent.

The integrated relative membership degree of each indicator for different risk grades can be normalized to uua, which is shown as follows:

$$uua=\left[\begin{array}{ccccc} 0 & 0.1414 & 0.4715 & 0.3254 & 0.0617\\ 0 & 0.0419 & 0.6469 & 0.3046 & 0.0066\\ 0 & 0.1741 & 0.4327 & 0.3018 & 0.0913\\ 0 & 0.0792 & 0.6084 & 0.2946 & 0.0178 \end{array}\right]$$

Use Equation (8) to calculate the grade characteristic value of this sample, as shown in Table 7.

Therefore, the flood disaster grade of Kezhou is the third, which is the same as the conclusion of Figure 1. From Table 7, we can see that although the model parameters are changed, the evaluation results are stable, which demonstrates the stability and effectiveness of the model. 

**Figure 3:**
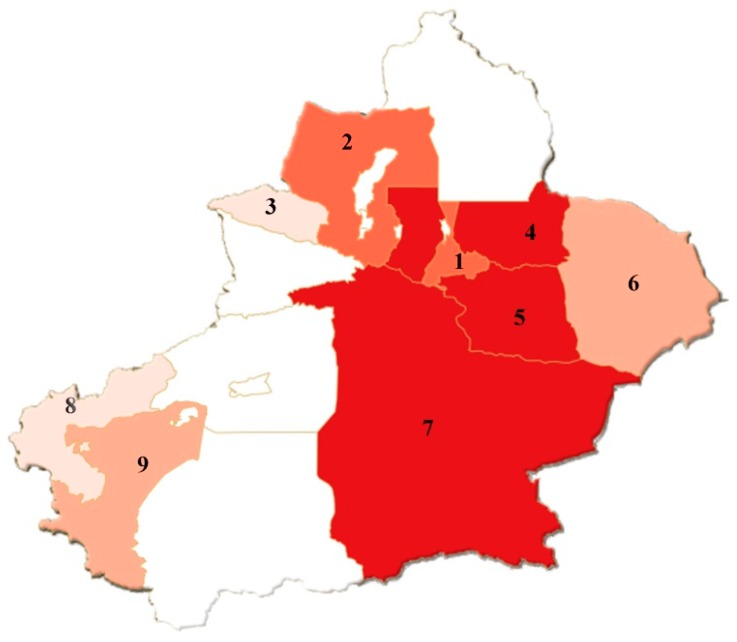
The distribution map of floods in the nine cities of Xinjiang.

Similarly, we get the same evaluation conclusions of flood disaster grades by the variable fuzzy recognition mode based on the other combined weights, which will be elaborated on in part 4.5. Calculating the grades in other regions in the same way, and the summary conclusions can be got in Table 8. The flood disaster level of each damage area can also be represented by a map, where different colors indicate corresponding disaster level. The distribution of floods in the nine cities of Xinjiang Uygur Autonomous Region can be seen in Figure 3. Scheme 10, Bingtuan, is not a regular administrative area, so it is not shown on the map.

### 4.5. Robustness Test

#### 4.5.1. Comparative Analysis of Evaluation Methods

This paper had the comprehensive evaluation results calculated by the multi-index evaluation model of variable fuzzy recognition, based on the combined weights according to a case. When changing the parameters of the variable fuzzy evaluation model, the results are all uniform, which demonstrates the stability of the variable fuzzy recognition model. In order to further verify the robustness of the model, we compared it with the principal component projection method [69] and the grey clustering method [21].

The results in Table 9 show that there are differences in flood disaster evaluation among the three methods. For this paper, the evaluation results of Changji, Turpan, Bazhou, and Bingtuan are all the same as Reference [69], but different from Reference [21]. According to the analysis of the original data, taking Changji as an example, we find that the four indicators in Changji, except “destroyed houses”, belonged to a range of extremely serious disasters are all in the interval of serious disaster and the indicator weight of destroyed houses is less than one third. Therefore, the evaluation result of the disaster grade, serious disaster, is reasonable and extremely serious disaster grade is too pessimistic. The evaluation results of this paper and Reference [69] are closer to the actuality than Reference [21].

For Hami and Kashgar, the evaluation results of this paper are different from the two comparative References [21,69]. Analyzing the original data once again, the four indicators in Hami, except damage area belonging to the range of moderate disaster, are all in the interval of serious disaster and the indicator weight of the damage area is relatively low. Therefore, the evaluation result of the disaster grade, serious disaster, is reasonable and the moderate disaster grade is unable to reflect the severity. In a similar way, the three indicators in Kashgar are all in the interval of serious disaster, only one falling in the range of moderate disaster. Therefore, the evaluation result of the disaster grade, serious disaster, is reasonable and moderate disaster grade is unable to reflect the severity.

According to the comparison of the results among different evaluation methods, we found that the projection values of the principal component projection method has a certain subjectivity, and the whitening function of the grey clustering method is unable to reflect the internal changes of disaster grades because of the theory’s limitation itself. The variable fuzzy recognition model, based on the combined weights, precisely describes the differences of the disaster losses among those evaluation objects in the form of a continuous real numbers, which makes the evaluation results more reliable and stable.

#### 4.5.2. Discussions of Weights

The variable fuzzy recognition model shows robustness well in multi-index decision-making for flood disaster as a result of the discussions in Section 4.5.1. Next, we will verify the rationality of the weights of this paper.

This paper uses the classic methods of the subjective and objective weights determination, combining them in proper order to form six kinds of combined weights according to the principle of minimum identification information, and then carries out the variable fuzzy evaluation. Simply applying the subjective weights, the evaluation results calculated by AHP, binary comparison method, and FCM are inconsistent. Since the subjective weights are easily affected by preferences and experiences from experts, the evaluation results are unreliable. While only applying the objective weights, different principle, and the mechanism of the entropy method and variable fuzzy method lead to different evaluation results in spite of the same original data. Because the objective weights are influenced by the accuracy of the iteration and the collected data, the evaluation results calculated are not creditable either. However, after the two kinds of objective weights, being corrected by the AHP or binary comparison method or FCM, the evaluation results, based on the combined weights, are all consistent. Therefore, simply relying on subjective weights or objective weights will not describe the evaluation results accurately, and the combined weights have a stronger explanatory ability and application validity. The results are summarized in Table 10.

As mentioned above, the multi-index evaluation model of variable fuzzy recognition based on the combined weights, having a good robustness, can be applied in the multi-index evaluation of flood disaster.

## 5. Conclusions

This paper established the multi-index evaluation model of variable fuzzy recognition for floods based on the combined weights. According to a specific case of Xianjiang in China, we not only verified the effectiveness and reliability targeting in the variable fuzzy recognition model itself, but also validated the rationality and robustness of the variable fuzzy recognition model based on the combined weights aiming at different weights and their combinations.

The variable fuzzy recognition model obtained four groups of evaluation values by means of changing parameter combinations, which improved the reliability and credibility of the evaluation results. When simply relying on the subjective weights, the evaluation results will be of great difference because of experts’ different preferences and experiences for indicators are not accurate enough. When only depending on the objective weights, the evaluation results can describe the internal relations of the objective data with high requirements on the accuracy of the data samples, and different calculation principles will lead to the instability of the evaluation results. Therefore, the combined weights considering both subjective and objective weights can not only reflect all the information of the decision matrix but can also avoid the distortion of the data by subjective experience, which makes the evaluation results more stable and rational, closer to the reality as much as possible.

The analysis of the case demonstrates that the model is simple and effective. The evaluation conclusions are reliable to be applied in the multi-index evaluation of flood disaster and provide reasonable suggestions for floods precaution and relief to management authorities.

However, there are still some limitations. Firstly, we only validate the robustness and rationality of the model itself, but lack adequate argument for the quality of the data and evaluation index system, which use an existing case from the reference. We should further explore much more actual flood data from other regions in China and conduct comparative analysis between regions. In addition, we will collect data of floods in Xinjiang, in different years, for comparative analysis on time series. Through the comparison of horizontal and vertical, the verification and application of the model proposed is more fully expanded. The establishment of the index system should be further discussed, and previous research and actual floods investigations will be comprehensively considered to ensure that the indicators can accurately evaluate the floods level. Secondly, although the floods evaluation model proposed in this study is an effective means for floods precaution and relief because of quickness, accuracy, and easiness, we can combine the traditional evaluation methods and those visualization technologies, such as remote sensing technology, to make real-time and dynamic evaluation for flood disaster. Additionally, we can use the machine learning method to predict floods and optimize indexes by social network analysis.

## Figures and Tables

**Table 1:** Saaty 1–9 matrix standard degree [60].

Comparison of Importance between $a_{i}$ and $a_{j}$	$a_{ij}$	$a_{ij}$
$a_{i}$ is as important as $a_{j}$	1	1
$a_{i}$ is slightly more important than $a_{j}$	3	1/3
$a_{i}$ is obviously more important than $a_{j}$	5	1/5
$a_{i}$ is intensely more important than $a_{j}$	7	1/7
$a_{i}$ is extremely more important than $a_{j}$	9	1/9
Importance of $a_{i}$ and $a_{j}$ falls in between the above grade	2,4,6,8	Corresponding reciprocal

**Table 2:** Average random consistency scale RI [60].

Rank	1	2	3	4	5	6	7	8	9	10	11	12
RI	0	0	0.58	0.90	1.12	1.24	1.32	1.41	1.45	1.49	1.52	1.54

**Table 3:** The flood loss statistics in different regions [68].

Region	Scheme	Damage Area/102 km^2^	Hit Population/104 Persons	Destroyed Houses/104 m^2^	Direct Economic Loss/107 RMB
Urumchi	1	0.1543	6.0000	20.6900	3.4800
Tacheng	2	1.3740	5.9700	6.2350	1.6080
Bozhou	3	0.2601	4.3500	2.8430	0.1770
Changji	4	2.3520	9.4000	54.5000	7.9100
Turpan	5	1.6673	2.9600	58.7280	4.9460
Hami	6	0.5458	2.6200	5.1050	1.8260
Bazhou	7	1.0792	4.5400	21.7130	7.8800
Kezhou	8	0.3410	5.6000	1.5560	0.3950
Kashgar	9	0.2140	20.000	1.8900	1.4300
Bingtuan	10	4.6026	24.2700	13.5920	6.3270

**Table 4:** The normalization of original data.

Scheme	Damage Area/102 km^2^	Hit Population/104 Persons	Destroyed Houses/104 m^2^	Direct Economic Loss/107 RMB
1	1.0000	0.8439	0.6653	0.5729
2	0.7258	0.8453	0.9182	0.8149
3	0.9762	0.9201	0.9775	1.0000
4	0.5059	0.6868	0.0740	0.0000
5	0.6599	0.9843	0.0000	0.3833
6	0.9120	1.0000	0.9379	0.7868
7	0.7921	0.9113	0.6474	0.0039
8	0.9580	0.8624	1.0000	0.9718
9	0.9866	0.1972	0.9942	0.8380
10	0.0000	0.0000	0.7895	0.2047

**Table 5:** The results of combined weights.

Weight Type	Entropy Method	Variable Fuzzy Method
AHP	(0.1341,0.2711,0.2218,0.3730)	(0.2540,0.3014,0.1854,0.2592)
Binary comparison method	(0.1903,0.2628,0.2148,0.3321)	(0.2937,0.2378,0.1786,0.2899)
FCM	(0.2052,0.2257,0.2734,0.2958)	(0.3146,0.2029,0.2258,0.2566)

**Table 6:** Classify standards of flood evaluation indicators.

Disaster Grades/Indicators	Damage Area/102 km^2^	Hit Population/104 Persons	Destroyed Houses/104 m^2^	Direct Economic Loss/107 RMB
Extremely serious disaster	(10, +∞)	(10, +∞)	(10, +∞)	(10, +∞)
Serious disaster	[1, 10]	[1, 10]	[1, 10]	[1, 10]
Moderate disaster	[0.1, 1]	[0.1, 1]	[0.1, 1]	[0.1, 1]
Low-grade disaster	[0.01, 0.1]	[0.01, 0.1]	[0.01, 0.1]	[0.01, 0.1]
Mild disaster	(−∞, 0.01)	(−∞,0.01)	(−∞, 0.01)	(−∞, 0.01)

**Table 7:** Evaluation results of different model parameters combination.

Combination Forms	Level Characteristic Value	Evaluation Grade
α=1,p=1	3.3074	III
α=2,p=1	3.2759	III
α=1,p=2	3.3104	III
α=2,p=2	3.2511	III

**Table 8:** Evaluation results of floods in different regions.

Parameter Values	1	2	3	4	5	6	7	8	9	10
α=1,p=1	3.8772	3.8350	3.2375	4.2999	4.0561	3.6118	4.1216	3.3074	3.7028	4.2942
α=2,p=1	3.9751	3.8207	3.2012	4.3578	4.0949	3.5899	4.1462	3.2759	3.6562	4.3758
α=1,p=2	3.8149	3.8400	3.2103	4.2824	4.0657	3.6090	4.1324	3.3104	3.7293	4.2449
α=2,p=2	3.9508	3.8023	3.1301	4.3570	4.1146	3.6242	4.1732	3.2511	3.7601	4.3402
Evaluation mean	3.9045	3.8245	3.1948	4.3243	4.0828	3.6087	4.1434	3.2862	3.7121	4.3138
Evaluation Results	IV	IV	III	IV	IV	IV	IV	III	IV	IV

**Table 9:** Comparison of the flood disaster grades by different methods.

Value of Parameters	1	2	3	4	5	6	7	8	9	10
Variable fuzzy method	IV	IV	IV	IV	IV	IV	IV	III	IV	IV
Principal component projection method	IV	IV	III	IV	IV	III	IV	III	III	IV
Gray clustering method	IV	IV	III	V	V	III	V	III	III	V

**Table 10:** Comparison of the flood disaster grades by different weight combinations.

Weight Type	Combination Type	1	2	3	4	5	6	7	8	9	10
Subjective weights	AHP	IV	IV	III	IV	IV	IV	IV	III	IV	IV
	Binary comparison	IV	IV	III	IV	IV	IV	IV	III	III	V
	FCM	IV	IV	III	IV	IV	IV	IV	III	IV	IV
Objective weights	Entropy method	IV	IV	III	IV	IV	IV	IV	III	IV	IV
	Variable fuzzy method	III	IV	III	IV	IV	III	IV	III	III	IV
Combined weights	AHP + Entropy method	IV	IV	III	IV	IV	IV	IV	III	IV	IV
	AHP + Variable fuzzy method	IV	IV	III	IV	IV	IV	IV	III	IV	IV
	Binary comparison + Entropy method	IV	IV	III	IV	IV	IV	IV	III	IV	IV
	Binary comparison + Variable fuzzy method	IV	IV	III	IV	IV	IV	IV	III	IV	IV
	FCM + Entropy method	IV	IV	III	IV	IV	IV	IV	III	IV	IV
	FCM+ Variable fuzzy method	IV	IV	III	IV	IV	IV	IV	III	IV	IV
